# Mcm5 Represses Endodermal Migration through Cxcr4a-itgb1b Cascade Instead of Cell Cycle Control

**DOI:** 10.3390/biom12020286

**Published:** 2022-02-10

**Authors:** Yu Zhang, Jiamin Xia, Min Liu, Bingyu Chen, Min Yang, Xiaoping Yu, Yu Ou, Shurong Li, Xindong Liu, Yi Feng, Bingyin Su, Sizhou Huang

**Affiliations:** 1 Development and Regeneration Key Laboratory of Sichuan Province, Department of Anatomy and Histology and Embryology, School of Basic Medical Sciences, Chengdu Medical College, Chengdu 610500, China; zhangyu_1023@126.com (Y.Z.); xiajiamin0709@126.com (J.X.); lium202201@126.com (M.L.); bingyuyu0825@126.com (B.C.); myang@cmc.edu.cn (M.Y.); 1000444@cmc.edu.cn (X.Y.); ouyu2022@126.com (Y.O.); lsrsus@126.com (S.L.); 2 Department of Neurology, The Second Affiliated Hospital of Chengdu Medical College, China National Nuclear Corporation 416 Hospital, Chengdu 610051, China; xd_liu2022@126.com; 3 Centre for Inflammation Research, Queen’s Medical Research Institute, University of Edinburgh, 47 Little France Crescent, Edinburgh EH16 4TJ, UK; yi.feng@ed.ac.uk

**Keywords:** *mcm5*, cell cycle, cxcr4a, endodermal migration, liver bifida

## Abstract

Minichromosome maintenance protein 5 (MCM5) is a critical cell cycle regulator; its role in DNA replication is well known, but whether it is involved in the regulation of organogenesis in a cell cycle-independent way, is far from clear. In this study, we found that a loss of *mcm5* function resulted in a mildly smaller liver, but that *mcm5* overexpression led to liver bifida. Further, the data showed that *mcm5* overexpression delayed endodermal migration in the ventral–dorsal axis and induced the liver bifida. Cell cycle analysis showed that a loss of *mcm5* function, but not overexpression, resulted in cell cycle delay and increased cell apoptosis during gastrulation, implying that liver bifida was not the result of a cell cycle defect. In terms of its mechanism, our data proves that *mcm5* represses the expression of *cxcr4a*, which sequentially causes a decrease in the expression of *itgb1b* during gastrulation. The downregulation of the *cxcr4a-itgb1b* cascade leads to an endodermal migration delay during gastrulation, as well as to the subsequent liver bifida during liver morphogenesis. In conclusion, our results suggest that in a cell cycle-independent way, *mcm5* works as a gene expression regulator, either partially and directly, or indirectly repressing the expression of *cxcr4a* and the downstream gene *itgb1b*, to coordinate endodermal migration during gastrulation and liver location during liver organogenesis.

## 1. Introduction

The minichromosome maintenance (MCM) 2–7 complex, the crucial component of the DNA replication licensing system [1], acts as the eukaryotic DNA replicative helicase during the cell cycle, in normal development and tumorigenesis [2,3]. Previous studies demonstrated that Mcm2–7 proteins are loaded onto DNA in 20-fold excess over the number of replication origins [4], but that normal replication is still maintained when Mcm2–7 proteins are decreased [5]. This raises the possibility that an additional role of MCM family members exists besides their role in normal DNA replication regulation. Indeed, early research has demonstrated that the excess of MCM complex, allows dormant origins of DNA replication when the cell is under replication stress [6]. Recently, a detailed analysis showed that most dormant DNA replication origins are within highly transcribed genes [7], implying that most of the components of ORC, including the members of the MCM family, are involved in gene expression regulation by binding with the genomic DNA within highly transcribed genes.

MCM5 is a member of the family of microchromosomal maintenance proteins and is highly conserved in eukaryotes [8]. During zebrafish development, *mcm5* is abundantly expressed in the proliferating cells [9]; *mcm5* loss of function results in overt cell cycle arrest and apoptosis in the head and eyes, suggesting the critical role of MCM during the cell cycle. Recently, we also found MCM5 to be involved in FMN migration [10], but the role of MCM5 in liver development was not addressed. In our current study, we used *mcm5* overexpression and loss of function to study the specific role of MCM5 in liver development. The data demonstrated that *mcm5* loss of function ubiquitously gave rise to mild cell cycle defect and apoptosis during gastrulation, but did not disrupt endodermal convergence to the dorsal side and the final liver location. On the contrary, *mcm5* overexpression led to liver bifida [11], where ‘liver bifida’ means both the left and right side of the embryo displays liver development (as is seen in cardia bifida [12]), resulting from delayed endodermal migration in the ventral–dorsal axis (V-D axis) during gastrulation. As its mechanism via a cell cycle- and cell apoptosis-independent way, *mcm5* repressed the transcription of *cxcr4a* and subsequently downregulated the expression of *itgb1b*, which finally delayed endodermal migration and induced liver bifida. Our study directly identifies the specific role of MCM5 in endoderm migration and in the final liver location as being partially through its regulation of the expression of *cxcr4a* and *itgb1b* but not via cell cycle control. Our results also indirectly explain why an excess of MCM complex is found within highly transcribed genes [7], where it should regulate the expression of critical genes to coordinate organ development.

## 2. Materials and Methods

### 2.1. Fish Maintenance 

Wild type (AB), transgenic line *Tg(fabp10:EGFP)*, *Tg(sox17:EGFP)* [13], *cxcr4a^mu20^* [13], and *mcm5* mutant [10] fishes were maintained in standard conditions at 28.5 °C. The developmental stages were characterized as previously described [14].

### 2.2. Plasmid Construction 

The total RNA was extracted following the manufacturer’s instructions (TRIzol, Ambion, Austin, TX, USA). cDNA was prepared using a Revert Aid First Strand cDNA Synthesis Kit (Fermentas, Waltham, MA, USA) according to the manufacturer’s instructions. *mcm5* and *cxcr4a* CDS were individually amplified by PCR (PrimeSTAR Max Premix, Takara, Kusatsu, Japan) and cloned into the vector PCS^2+^ to generate the expression constructs (5x In-Fusion HD Enzemy Premix, Takara). 

### 2.3. Generation of the Tg(Hsp70l:mcm5-T2A-mCherry) Transgenic Line

To generate the *Tg(Hsp70l:mcm5-T2A-mCherry)* transgenic line, first, the transgenic plasmid was produced via the method described in [15]. After constructing the plasmid, it was co-injected with I-SecI and I-SecI buffer in the cell body at the one-cell stage. When the embryos grew to 4 dpf, those embryos displaying eye fluorescein were screened out and grown for alternate experiments (Founder, Fo), and the remaining embryos were then crossed with a wild-type fish (AB strain) to produce the F1 embryos. 

### 2.4. MO and mRNA Injection 

Morpholino oligos (MO) for *mcm5* (ATG MO, 5′-ATAGTTTCGATAAGTGCTGTC GATG-3′), *cxcr4*a (ATG MO, 5′-GGTGTTTGATTGTCTGACCTTCATG-3′), and control MO (5′-CCTCTTACCTCAGTTACAATTTATA-3′) were obtained from Gene Tools [9,11,16]. *mcm5* mRNA, *mCherry* mRNA, and *cxcr4a* mRNA were synthesized in vitro using the mMESSAGE mMACHINE Kit (AM1340, Ambion). The concentration of MO was as follows: *mcm5* MO, 300 µM; *cxcr4a* MO, 500 µM; control MO, 500 µM. The concentration of the mRNA injection was as follows: *mcm5* mRNA, 60 ng/µL; *mCherry* mRNA, 65 ng/µL; *cxcr4a* mRNA, 15 ng/µL. All of the MOs and mRNAs were injected at the 1–4-cell stage. 

### 2.5. Real-Time qRT-PCR and Primers

Real-time q-PCR was performed using Brilliant III Ultra-Fast SYBR Green QPCR Master Mix (Agilent Technologies, Santa Clara, CA, USA) and the CFX96 Real-Time System (BIO-RAD, Hercules, CA, USA), according to the manufacturers’ instructions. A transcription of beta-actin was used for normalization. The primers are listed in Table S1.

### 2.6. Whole-Mount In Situ Hybridization

In situ hybridization was performed as described previously [17]. The probes *sox17*, *spaw*, *lft1*, *fabp10*, and *papc* were used [17]. The CDs of *vmhc* and *mcm5* were amplified via PCR and cloned into the pGEM-T easy vector system; then the plasmid was linearized, and the *vmhc* antisense probe and the *mcm5* antisense probe were synthesized. To synthesize probes *yap1*, *wwtri*, *itgb1b*, *itgb1a*, *cxcr4a*, and *cxcl12b*, the CDs for individual genes were amplified via PCR (an additional T7/Sp6 promoter was added to the start end of the Reverse Primer for each gene); part of the PCR product was used for sequencing to evaluate whether the right PCR product was obtained, while the remainder was used as a template to synthesize the probes [17]. 

### 2.7. Immunostaining

The embryos were fixed overnight with PEM at 4 °C, washed with PBS (5 min, 3x), and blocked with PBTN (4% BSA, 0.02%NaN_3,_ in PT) for 2 h at 4 °C. Then, the H3p primary antibody was diluted with PBTN to 1:100 and incubated on a shaker at 4 °C overnight. The embryos were washed with PT (0.3% Triton-X-100, in 1X PBS) for at least 20 min, 8 times. The secondary antibody (GeneTex 26800, Irvine, CA, USA) was diluted with PBTN to 1:500 and incubated overnight at 4 °C in the dark. Embryos were prepared for imaging by washing with PT for 30 min, at least 8 times.

### 2.8. Cell Apoptosis Staining

Embryos were dechorionated and fixed in 4% paraformaldehyde overnight at 4 °C, then washed with PBST buffer three times and permeabilized with methanol overnight. After washing three times with PBST (5 min each time), an In Situ Cell Death Detection Fluorescein kit (Roche 11684795910, Basle, Switzerland) was used to examine cell apoptosis according to the manufacturer’s instructions.

### 2.9. Cell Cycle Analysis

Twenty embryos were used for cell cycle analysis for each group. Briefly, embryos were washed with pre-cooled PBS, incubated, and blown in 1 mL of PBS with 0.25% trypsin to obtain single-cell suspensions. The cell suspensions were passed through a cell strainer (FALCON, NO.352340, Corning, NY, USA), followed by centrifugation at 4 °C for 2 min at 1000 g and the careful removal of the supernatant (2 times). The cells were resuspended in 0.25 mL of PBS and fixed with 0.75 mL of cold ethanol at 4 °C overnight. Then, the fixed cells were precipitated and resuspended in 500 µL of propidium iodide solution (0.1 mg/mL propidium iodide, 0.1% sodium citrate, 100 lg/mL RNase A, and 0.0002% Triton X-100), followed by analysis using a NovoSampler Q NO.2020098.

### 2.10. Statistical Analysis 

All data were analyzed using Novoexpress, ImageJ, and statistical software in Graphpad Prism 8 for Windows (GraphPad Software). Data are represented with significance values (*p*) denoted by * *p* < 0.05, ** *p* < 0.01, and *** *p* < 0.001. Experiments were performed at least three times for each condition.

## 3. Results

### 3.1. Mcm5 Overexpression Gives Rise to Liver Bifida

**Figure 1:**
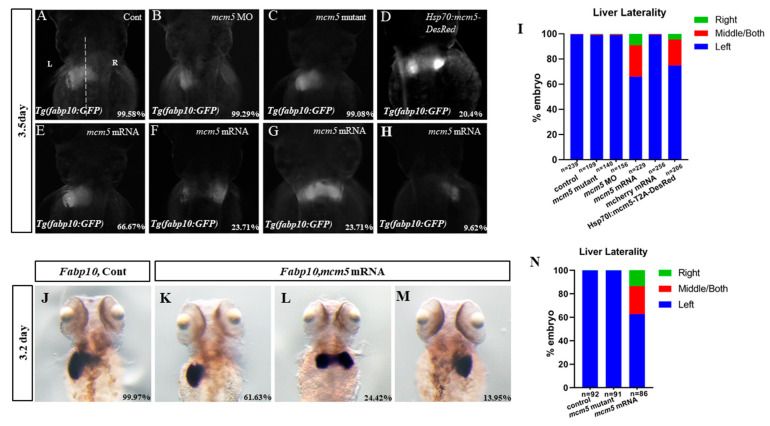
MCM5 overexpression, but not loss of function, leads to liver bifida. (**A**–**I**) In our observations, 99.58% of the *Tg(lfabp10:GFP)* control embryos displayed a left-sided liver (**A**,**I**, *n* = 293). In the *mcm5* morphants or *mcm5* mutants, the liver became mildly smaller (**B**,**C**) than that in the control embryos; 99.29% of the *mcm5* morphants (**B**,**H**, *n* = 140, *p* > 0.05) and 99.08% of the *mcm5* mutants (**C**,**I**, *n* = 103, *p* > 0.05) displayed a left-sided liver. In embryos with a forced expression of *mcm5*, the proportions of embryos with a left-sided liver, liver bifida, and a right-sided liver were 66.67% (**E**,**I**, *n* = 156, *p* = 0.00025), 23.71% (**F**,**G**,**I**, *n* = 156, *p* = 0.0026), and 9.62% (**H**,**I**, *n* = 156, *p* = 0.0064), respectively. Similarly, in embryos with a forced expression of *mcm5* by heat-shock at 40% epiboly, the proportion of embryos with liver bifida and a right-sided liver was increased when compared with the control embryos (**D**,**I**, *n* = 206, *p* = 0.0035). In total, 97.4% of the embryos injected with *mCherry* mRNA displayed a left-sided liver (**I**, *n* = 229, *p* = 0.35). (**J**–**N**) In the wild-type embryos, the rate of left-sided expression of *lfapb10* was 99.97% (**J**,**N**, *n* = 92), while 99.89% of the *mcm5* mutants displayed left-sided expression of *lfabp10* (**J**, *n* = 91, *p* = 0.93). In total, 61.63% (**K**,**N**, *n* = 86, *p* = 0009), 24.42% (**L**,**N**, *n* = 86, *p* = 0.0006), and 13.95% (**M**,**N**, *n* = 86, *p* = 0.0017) of embryos injected with *mcm5* mRNA displayed a left-sided, both-sided, and right-sided expression of *lfabp10*, respectively.

Since the quantity of MCM5 is largely more than that required for DNA replication [1], and *mcm5* is also expressed ubiquitously in proliferating cells, including in the liver of zebrafish [9] (Figure S1), we propose that MCM5 plays an extra role beside DNA duplication regulation during liver development. Since in *mcm5* morphants, the translation of the maternal *mcm5* mRNA would be knocked down [16], to fully address the role of MCM5 in liver development, we analyzed the phenotypes in our *mcm5* mutant [10], *mcm5* morphants, and those embryos overexpressing *mcm5* mRNA. The general phenotype in the *mcm5^−/−^* embryos was similar to that in a previously published *mcm5^−^*^/*−*^ allele [9]; the eyes and heads were smaller, and the length of embryo bodies was shorter (Figure S1). Regarding liver development, the *mcm5* morphants and the *mcm5* mutants displayed a mildly smaller liver than the control at 3 dpf (Figure 1A–C; Figure S2). When *mcm5* was overexpressed (Figure S3), a large number of embryos displayed liver bifida (Figure 1E–I). To confirm the specific role of *mcm5* overexpression, the transgenic line *Tg(Hsp70l:mcm5-T2A-desRed)* was generated (Figure S4) and was used to induce the expression of *mcm5* RNA via heat-shock at 40% epiboly (Figure S5); the phenotype of liver bifida was also observed in the treated embryos (Figure 1D,I). In contrast, in the controls, an injection of *mCherry* mRNA (65 pg) (Figure S6) did not lead to liver bifida (Figure 1I). To rule out the influence of the transgenic background of the embryos in these experiments, the expression of *fabp10* in the wild-type embryos, the *mcm5^−/−^* embryos, and the embryos injected with *mcm5* mRNA was examined. The data showed that embryos injected with *mcm5* mRNA displayed liver bifida and liver reversal (Figure 1K–N), but no liver localization defects were observed in the *mcm5^−/−^* embryos or in the controls (Figure 1 J,N). In addition, no heart bifida was observed in embryos injected with *mcm5* mRNA (Figure S7A–C). These findings show that *mcm5* overexpression specifically results in liver bifida. 

### 3.2. MCM5 Represses Endodermal Migration during Gastrulation

**Figure 2:**
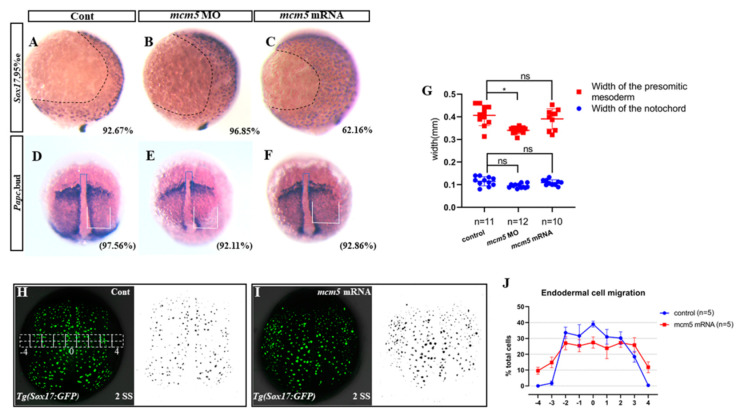
A forced expression of *mcm5* depressed endodermal migration. (**A**–**C**) Compared with that in the control (**A**, *n* = 109), the migration of endodermal cells in *mcm5* morphants was normal (**B**, *n* = 127), but the endodermal migration in embryos injected with *mcm5* mRNA was slowed down (**C**, *n* = 111, *p* = 0.0014). (**D**–**G**). *Papc* staining showed no difference in the width of the notochord (blue frame shown) between the control (**D**, *n* = 41; **G**, *n* = 11) and embryos injected with *mcm5* mRNA (**E**, *n* = 38; **G**, *n* = 10, *p* = 0.3), but the width of the notochord was mildly decreased in *mcm5* morphants (**E**, *n* = 38; **G**, *n* = 12, *p* = 0.015). The width of the presomitic mesoderm (white frame shown) in *mcm5* morphants was also mildly decreased (**E**, *n* = 38; **G**, *n* = 12, *p* = 0.12), but it was normal in embryos injected with *mcm5* mRNA (**F**, *n* = 56; **G**, *n* = 10, *p* = 0.69). (**H**–**J**) In *Tg(Sox17:GFP)* embryos, quantitative analysis showed no GFP-labeled cells in the “−4” area or “4” area in control embryos (**H**,**J**, *n* = 5), while about 10 and 12 GFP-labeled cells were observed in the “−4” area (**I**,**J**, *n* = 5, *p* = 0.00001) and the “4” area (**I**,**J**, *n* = 5, *p* = 0.00007), respectively, in embryos injected with *mcm5* mRNA.

During gastrulation, the liver progenitors migrate to the dorsal midline under the mesoderm and are then established as the left-sided liver at a late stage [18]. Any disturbance to the endodermal migration [11] or left–right asymmetric cascade [17,19] will cause randomized endodermal organ location, including liver bifida and liver reversal. To identify why liver bifida occurs in embryos overexpressing *mcm5* mRNA, the KV morphogenesis, midline, *Nodal/spaw*, and endodermal progenitor migration were examined. No differences were observed regarding the expression of *sox17*, *Nodal/spaw*, and *lft1* between treated embryos and the controls (Figure S8); however, endodermal migration (Figure 2A–C), but not mesodermal migration (Figure 2D–G), was slower at the V-D axis in embryos injected with *mcm5* mRNA. Further, the location of the GPF-labelled cells in *Tg(sox17:GFP)* embryos was analyzed at the two-somite stage (Figure 2H–J). In control embryos, no GFP-labelled cells were localized in the regions “−4” and “4” (Figure 2H,J, blue line shown), but about 10 and 12 GFP-labelled cells were localized in regions “−4” and “4”, respectively, in embryos injected with *mcm5*mRNA (Figure 2I,J, red line shown). These results further confirmed that endodermal migration was reduced in embryos overexpressing *mcm5* mRNA, and this is possibly the reason for liver bifida during liver morphogenesis.

### 3.3. Overexpression of mcm5 did Not Result in Cell Cycle Progress Defect

**Figure 3:**
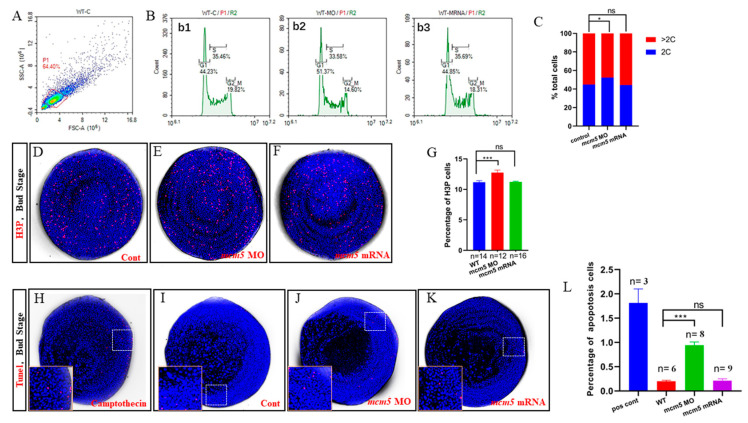
*mcm5* loss of function, but not overexpression, leads to cell cycle arrest and cell apoptosis at the bud stage. (**A**–**C**) Flow cytometry analysis showed that 44.23% of cells in control embryos were in the G1 phase at the bud stage (**Bb1**,**C**, *n* = 3), 51.37% of cells in *mcm5* morphants were in the G1 phase (**Bb2**,**C**, *n* = 3, *p* = 0. 023), and 44.85% of cells in embryos injected with *mcm5* mRNA were in the G1 phase (**Bb3**,**C**, *n* = 3, *p* = 0.1). Specifically, 44.2%, 35.4%, and 19.8% of cells in control embryos stayed in the G1 phase, S phase, and G2/M phase, respectively (**Bb1**); 51.3%, 33.5%, and 14.6% of cells in *mcm5* morphants stayed in the G1 phase, S phase, and G2/M phase, respectively (**Bb2**); 44.8%, 35.6%, and 18.3% of cells in embryos injected with *mcm5* mRNA stayed in the G1 phase, S phase, and G2/M phase, respectively (**Bb3**). (**D**–**G**) The proportion of H3p positive cells in the control embryos, *mcm5* morphants, and embryos injected with *mcm5* mRNA were 11.19% (**D**,**G**, *n* = 14), 12.76% (**E**,**G**, *n* = 12, *p* = 0.0067), and 11.23% (**F**,**G**, *n* = 16, *p* = 0.75), respectively. (**H**–**L**) The proportion of apoptotic cells in the camptothecin-treated embryos, control, *mcm5* morphants, and embryos injected with *mcm5* mRNA were 1.75% (**H**,**L**, *n* = 3, *p* = 0.000024), 0.20% (**I**, **L**, *n* = 6), 0.76% (**J**,**L**, *n* = 8, *p* = 0.0042), and 0.22% (**K**,**L**, *n* = 9, *p* = 0.39), respectively.

The cell cycle and cell differentiation must be coordinated properly during organogenesis [20], as disrupting the cell cycle results in cell fate determination defects [21]. Therefore, we evaluated the causation between the cell cycle and endodermal migration in embryos injected with *mcm5* mRNA. Specifically, 44.2%, 35.4%, and 19.8% of cells in the control embryos stayed in the G1 phase, S phase, and G2/M phase, respectively (Figure 3(Bb1)); 51.3%, 33.5%, and 14.6% of cells in *mcm5* morphants stayed in the G1 phase, S phase, and G2/M phase, respectively (Figure 3(Bb2)); 44.8%, 35.6%, and 18.3% of cells in embryos injected with *mcm5* mRNA stayed in the G1 phase, S phase, and G2/M phase, respectively (Figure 3(Bb3)). In total, 55.77% of cells stayed in the S/G2/M phase at the bud stage in control embryos (Figure 3B,C); in *mcm5* morphants and *mcm5*-overexpressing embryos, 48.63% and 55.15% of cells, respectively, stayed in the S/G2/M phase (Figure 3B,C). This result indicates that *mcm5* loss of function, but not *mcm5* overexpression, led to more cells staying in the G1 phase, meaning that only *mcm5* loss of function results in mild cell cycle delay. Since cell cycle delay results in decreased cell proliferation and increased cell apoptosis [16],we checked whether cell proliferation and cell apoptosis were disturbed in the treated embryos. Immunostaining experiments showed that the number of H3P-positive cells in *mcm5* morphants was increased (Figure 3E,G), while the number of H3P-positive cells in *mcm5*-overexpressing embryos (Figure 3F,G) was similar to that in the control (Figure 3 D,G). Meanwhile, the proportions of apoptotic cells in control embryos and in embryos treated with camptothecin, *mcm5* MO, and *mcm5* mRNA were 0.25%, 1.75%, 0.85%, and 0.27%, respectively, meaning that only *mcm5* morphants—and not embryos overexpressing *mcm5* mRNA, displayed increased cell apoptosis (Figure 3H–L). These two sets of experiments showed that the liver bifida observed in embryos injected with *mcm5* mRNA was not the result of cell cycle delay. 

### 3.4. Cxcr4a-itgb1b Cascade Mediates MCM5 to Regulate Endodermal Migration

**Figure 4:**
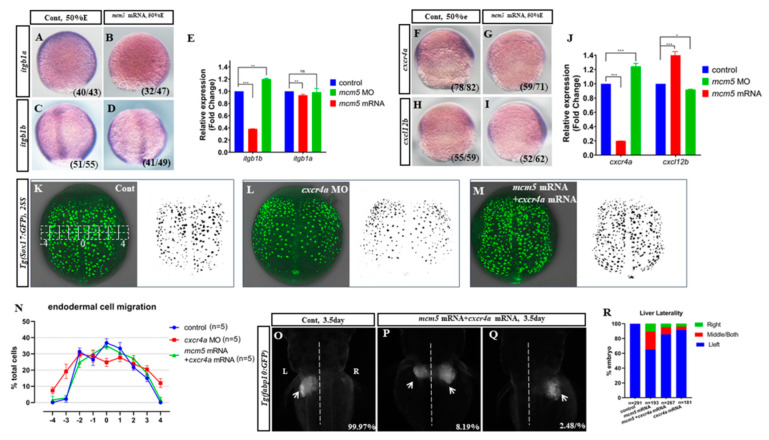
The downregulation of the Cxcr4a-itgb1b cascade slowed down endodermal migration in embryos injected with *mcm5* mRNA. (**A**–**E**) Compared with that in control embryos (**A**,**C**), the expression of *itgb1a* and *itgb1b* was decreased in embryos injected with *mcm5* mRNA (**B**,**D**). Quantitative PCR results showed that the expression level of *itgb1a* mRNA in embryos injected with *mcm5* mRNA was 0.9-fold to that in the control (**E**, *p* = 0.001), but it was no different in the *mcm5* morphants or in the control (**E**, 0.95-fold, *p* = 0.67). The *itgb1b* level in embryos injected with *mcm5* mRNA was 0.38-fold to that in the control (**E**, *p* = 0.001), while the level in *mcm5* morphants was 1.20-fold to that in the control (**E**, *p* = 0.03). (**F**–**J**) In embryos injected with *mcm5* mRNA, the expression levels of *cxcr4a* and *cxcl12b* were decreased (**F**,**G**) and increased (**H**,**I**), respectively. Quantitative PCR indicated that the expression levels of *cxcr4a* and *cxcl12b* in embryos injected with *mcm5* mRNA were also downregulated (**J**, 0.19-fold relative to the control, *p* = 0.0009) and upregulated (**J**, 1.35-fold relative to the control, *p* = 0.0002), respectively. In *mcm5* morphants, the expression levels of *cxcr4a* and *cxcl12b* were upregulated (**J**, 1.24-fold relative to the control, *p* = 0.0003) and downregulated (**J**, 0.9-fold relative to the control, *p* = 0.02), respectively. (**K**–**M**) Compared with that in the control (**K**, *n* = 25, 92%), GFP-labeled endodermal migration in *cxcr4a* morphants was slowed down (**L**, *n* = 122, 88.5%, *p* < 0.01), but *mcm5* mRNA and *cxcr4a* mRNA co-injection restored endodermal migration (**M**, *n* = 98, 73.5%, *p* < 0.05). Quantitative analysis for the selected area (shown in **K**) showed that migration of the delayed endodermal cells in the “−4” area and the “4” area in embryos injected with *mcm5* mRNA was restored by co-injection with *cxcr4a* mRNA (**N**, *n* = 5). (**O**–**R**) An injection of *cxcr4a* mRNA restored the liver location in embryos injected with *mcm5* mRNA. An injection of *cxcr4a* mRNA (30 ng/µL or more) resulted in a small proportion of the embryos displaying liver bifida (R, last column shown, *n* = 181, *p* < 0.05). Co-injection with a low dose of *cxcr4a* mRNA (15 ng/µL) and mcm5 mRNA resulted in 10.1% and 4.7% of embryos displaying liver bifida and a right-sided liver, respectively (**P**,**Q**). Co-injection with *cxcr4a* mRNA and *mcm5* mRNA restored the liver location phenotype in embryos injected with *mcm5* mRNA (**R**, columns 2 and 3, *n* = 267, *p* < 0.05).

Hippo signaling and the *cxcr4a*-*itgb1b* cascade were reported to be involved in regulating endoderm cell migration during gastrulation [11,22,23]. In situ and q-PCR experiments showed that *Yap1*, *wwtr1*, *last2*, and *ctgfa* were not significantly affected in embryos injected with *mcm5* mRNA (Figure S9), while the expression levels of *itgb1a* and *itgb1b* were decreased (Figure 4A–D), especially that of *itgb1b* (Figure 4E). In addition, the downregulation of the MCM5 function by means of a *mcm5* MO injection increased the transcription of *itgb1b* (Figure 4E). These data showed the possibility that *mcm5* negatively regulates *itgb1b* transcription during gastrulation. Next, we examined the expression of *cxcl12b/cxcr4a* in embryos injected with *mcm5* mRNA. Interestingly, *cxcr4a* was greatly downregulated in the *mcm5*-mRNA-injected embryos (Figure 4 F,G,J), but *cxcl12b* was mildly upregulated (Figure 4H–J). This result was confirmed by the observation that the expression levels of *cxcr4a* and *cxcl12b* were increased and decreased, respectively, in *mcm5* morphants (Figure 4J). These results suggest the possibility that the downregulated *cxcr4a-itgb1b* delayed endodermal migration at the V-D axis when *mcm5* was overexpressed. To investigate this hypothesis, we examined whether restoring the *cxcr4a* function could rescue the phenotype in the embryos injected with *mcm5* mRNA. We titrated a concentration of *cxcr4a* mRNA and co-injected *mcm5* mRNA and *cxcr4a* mRNA (15 ng/µL) into *Tg(Sox17:GFP)*/*Tg(fabp10::GFP)* transgenic embryos; then, we analyzed the endodermal migration and the liver location. At the two-somite stage, an *cxcr4a* MO injection greatly delayed endodermal migration ([23], Figure 4L), and an injection of *cxcr4a* mRNA, rescued endodermal migration delay in embryos injected with *mcm5* mRNA (Figure 4M,N). Further, liver bifida and reversed liver rates were decreased (Figure 4P,Q). These data suggest that *cxcr4a* rescued the liver bifida phenotype in embryos overexpressing *mcm5*, and that *cxcr4a*, at least partially, mediated *mcm5* to regulate endodermal migration during gastrulation.

## 4. Discussion

It is well known that the MCM2–7 complex acts as the eukaryotic DNA replicative helicase during DNA replication in normal development and tumorgeneis [2,3]. As one component of the MCM2–7 complex, MCM5 also plays a critical role in the unwinding of the duplex DNA during DNA replication [24]. In early zebrafish development, the loss of *mcm5* function disturbs the cell cycle, and gives rise to cell apoptosis and endomesodermal delamination defects, thus clarifying the causality between the cell cycle and cell fate determination [16]. This result confirms that the specific role of MCM5 during early embryonic development is a cell cycle regulatory role. On the other hand, previous studies demonstrated that Mcm2–7 proteins are loaded onto DNA in 20-fold excess over the number of replication origins [4]. These facts support the hypothesis that *mcm5* has an additional critical role besides regulating DNA replication within proliferating cells. Indeed, recent research suggests that most of the extra MCM family members are within highly transcribed genes [7], which implies a possible role of MCM family members (including MCM5) in gene transcription regulation. In our current study, we found that *mcm5* loss of function resulted in a mildly smaller liver at 3.5dpf. Further, overexpression of *mcm5* induced by the injection of *mcm5* mRNA led to liver bifida (Figure 1F,G). To rule out the non-specific role of an injection of *mcm5* mRNA, *mCherry* mRNA was synthesized and the transgenic line *Tg(Hsp70l:mcm5-T2A-desRed)* was generated. An injection of *mCherry* mRNA didn’t lead to liver bifida but heat-shock induced overexpression of MCM5, resulting in liver bifida (Figure S5; Figure 1D,I) similar to the phenotype seen in embryos injected with MCM5 mRNA. These results partially prove that overexpression of MCM5 leads to liver bifida.

To confirm the role of MCM5 in liver development, we tried to find out how overexpression of MCM5 specifically induces liver bifida. The data showed that *mcm5* loss of function upregulated the expression of *cxcr4a* and *itgb1b,* but did not upregulate the expression of *itgb1a* (Figure 4J,E). In addition, we did not find an obvious liver developmental phenotype in the embryos with a loss of *mcm5* function (Figure 1B,C). On the contrary, the overexpression of *mcm5* downregulated the expression of *cxcr4a* and *itgb1b* (Figure 4J,E) and subsequently gave rise to delayed endodermal migration (Figure 2C,H–J) and liver bifida (Figure 1E–H). Also, the expression of *itgb1a* was not significantly changed in the treated embryos. To further confirm that the downregulation of *cxcr4a-itgb1b* results in liver bifida in embryos overexpressed with *mcm5*, an injection of *cxcr4a* mRNA was used to rescue the liver bifida phenotype in embryos overexpressed with *mcm5*. The results showed that *cxcr4a* mRNA partially rescues the liver phenotype in embryos overexpressed with *mcm5* (Figure 4M,O–R). These three sets of experiments suggest the possibility that *mcm5* negatively regulates the expression of *cxcr4a* and *itgb1b*. Of note, our data didn’t identify the detailed mechanism of how *mcm5* regulates *cxcr4a* expression. Consequently, far more research work is required to clarify this mechanism, which will help in our understanding of how *mcm5* specifically regulates the expression of *cxcr4a*.

To exclude the possibility that the forced expression of *mcm5* mRNA leads to cell cycle delay and cell apoptosis, indirectly delaying endodermal migration, we evaluated the cell cycle situation in embryos injected with *mcm5* mRNA. The injection of *mcm5* mRNA did not give rise to cell cycle delay (Figure 3B,C,F,G) or cell apoptosis (Figure 3K,L), thereby excluding this possibility. On the contrary, *mcm5* loss of function (injection with *mcm5* MO) resulted in cell cycle delay and increased apoptosis (Figure 3J,L), which is consistent with our early research [16]. Thus, according to our previous study and our current data, we propose a hypothesis and model of how *mcm5* regulates organ development (Figure S10). MCM5 works as a dual-function factor during early development. During gastrulation, most of the cells are in the cell cycle with high proliferation potentiality. On the one hand, ORC acts to recruit Cdc6 and Cdt1 to the DNA replication origins, which together to recruit the MCM complex to the origins for the pre-RC formation [25]. Here, *mcm5* works as a subunit of the hexameric helicase to unwind the DNA helix, subsequently initiating DNA replication, thereby ensuring that DNA integrates during cell proliferation (as shown in role “1”). On the other hand, in a cell-cycle independent manner, it is possible that *mcm5* works as a critical transcription regulator to repress *cxcr4a* expression, which subsequently regulates *itgb1b* expression and endodermal migration (as shown in role “2”). This model only clarifies one role of *mcm5* in regulating gene expression; far more of *mcm5′*s function awaits discovery through future research. 

Recently, the role of cell cycle regulators in embryonic development and disease has been widely reported, including for the members of the MCM family [2,26,27,28]. In mice, mutations in MCM2-MCM7 cause genomic instability and render female mouse embryos markedly more susceptible to embryonic lethality [29]; this role is dependent on DNA replication and the cell cycle. In zebrafish, *mcm2* was found to work as the target gene of *foxn1* to regulate thymic epithelial cell proliferation [30]; this study only identified *mcm2* as the proliferation regulator during thymus development, in a manner that is also cell cycle-dependent. More recently, Philipp’s group discovered that MCM2 binds to the transcription start sites of cilia-inhibiting genes to block RNA polymerase II-mediated transcription [31], to coordinate ciliogenesis, and the subsequent organ LR patterning. Their work also showed that MCM7 is involved in ciliogenesis via a subset of genes and pathways distinct from those of MCM2 [31], implying different roles of individual members of the MCM family in embryonic development. In our study, we identified the possibility that *mcm5* works as a transcription regulator, coordinating endodermal migration by repressing the activity of the Cxcr4a-Itgb1b cascade, instead of via cell cycle control. While regulating gene transcription, MCM3 is reported to be associated with MCM5, and this interaction is required for the MCM5/Stat1 complex to activate the downstream gene transcription [32]. Accordingly, it is possible that *mcm5* acts as a part of a complex to regulate *cxcr4a* transcription. If MCM5 works as part of a complex, manipulation of the expression of the complex’s elements would also have the same function. This hypothesis needs far more investigation to be clarified and confirmed. In addition, far more research is required to clarify whether the role of *mcm5* is direct or indirect.

## Data Availability

All the data is in the manuscript and supplementary materials.

## Supplementary Materials

The following are available online at https://www.mdpi.com/article/10.3390/biom12020286/s1, Figure S1: Expression of *mcm5* in wild-type and *mcm5* mutant embryos at different stages. Figure S2: Liver phenotype in *mcm5* mutants and *mcm5* morphants. Figure S3: The overall phenotypes of the embryos injected with different mRNA. Figure S4: The construction of *Tg(Hsp70l:mcm5-T2A-desRed)* and overexpression of *mcm5* induced by heat-shock. Figure S5: Heat-shock induced the expression of *mcm5* in *Tg(Hsp70l:mcm5-T2A-mCherry)* embryos. Figure S6: The expression of *mCherry* after injection of *mCherry* mRNA. Figure S7: *mcm5* mRNA injection did not lead to heart bifida. Figure S8: The expression of left–right-patterning-related genes. Figure S9: Yap signaling was not disturbed greatly in embryos injected with *mcm5* mRNA. Figure S10: The model for *mcm5* regulating DNA replication and endodermal migration in early zebrafish development. Table S1: Primers for q-PCR.
