# Supplementary material for: Mcm5 Represses Endodermal Migration through Cxcr4a-itgb1b Cascade Instead of Cell Cycle Control
Source: Biomolecules. 2022 Feb 10;12(2):286. doi: 10.3390/biom12020286 (PMC8961633; doi:10.3390/biom12020286)
Supplement: Supplementary file 1 [file biomolecules-12-00286-s001.zip › biomolecules-1490489-supplementary.pdf]

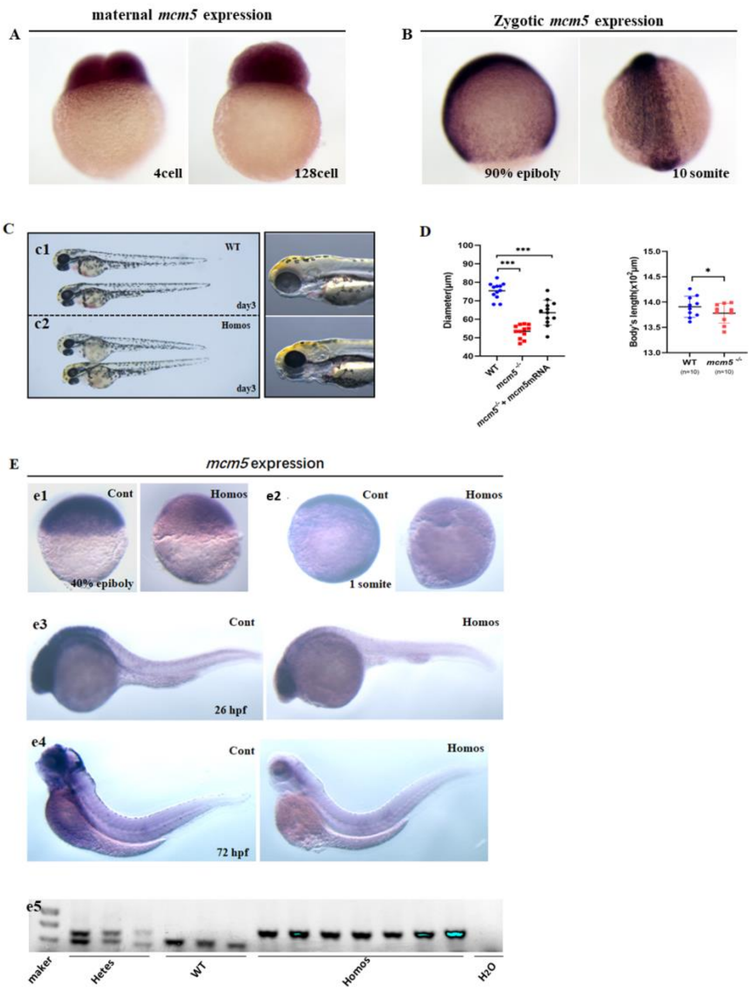

Figure S1. Expression of *mcm5* in wild type and *mcm5* mutant embryos at different stages. (**A**–**B**) The expression of *mcm5* in early developmental stages. *mcm5* is maternally expressed (**A**) and it is expressed in proliferating cells (**B**). (**C**–**D**) The living embryos from wild type cluster and *mcm5* homozygotes. The heads/eyes in *mcm5* mutants were smaller than that in wild type embryos (**D**, $n = 12$， $p = 0.0283$), and the body length was slight shorter in *mcm5* mutants (**D**, $n = 10$， $p < 0.0001$). (Ee1-e5) The expression of *mcm5* mRNA in wild type and mutant embryos. The expression of *mcm5* in wild type embryos and *mcm5* mutants at 40% epiboly (Ee1, control embryos, $n = 32$; homozygotes, $n = 7$), 1 somite stage (Ee2, control embryos, $n = 17$; homozygotes, $n = 6$), 26 hpf (Ee3, control embryos, $n = 15$; homozygotes, $n = 6$) and 72 hpf (Ee4, control embryos, $n = 26$; homozygotes, $n = 8$). The wild type and *mcm5* mutant embryos were examined by genome typing (Ee5, Control embryos, $n = 12$; homozygotes, n=5). *$p < 0.05$,***$p < 0.0001$.

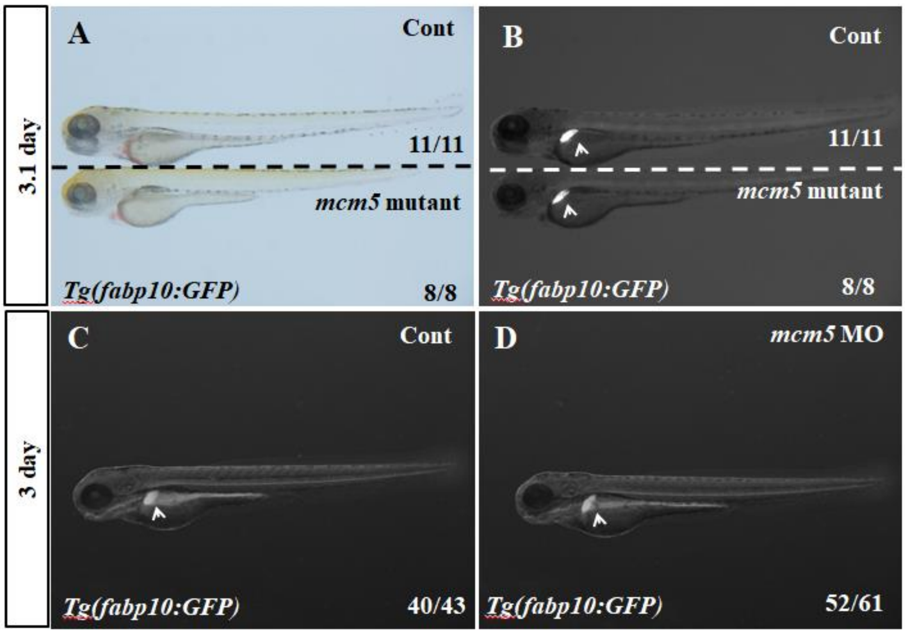

Figure S2. Liver phenotype in *mcm5* mutants and *mcm5* morphants. (**A**–**B**) Comparing with the control embryos (**A**, **B**, up embryos), the *mcm5* mutant embryos displayed smaller eye and head (**A**, down embryo), mild smaller liver (**B**, down embryo, arrow head shown). (**C**–**D**) The liver in *mcm5* morphants (**D**, arrow head) was mild smaller than that in control morphants (**C**, arrow head).

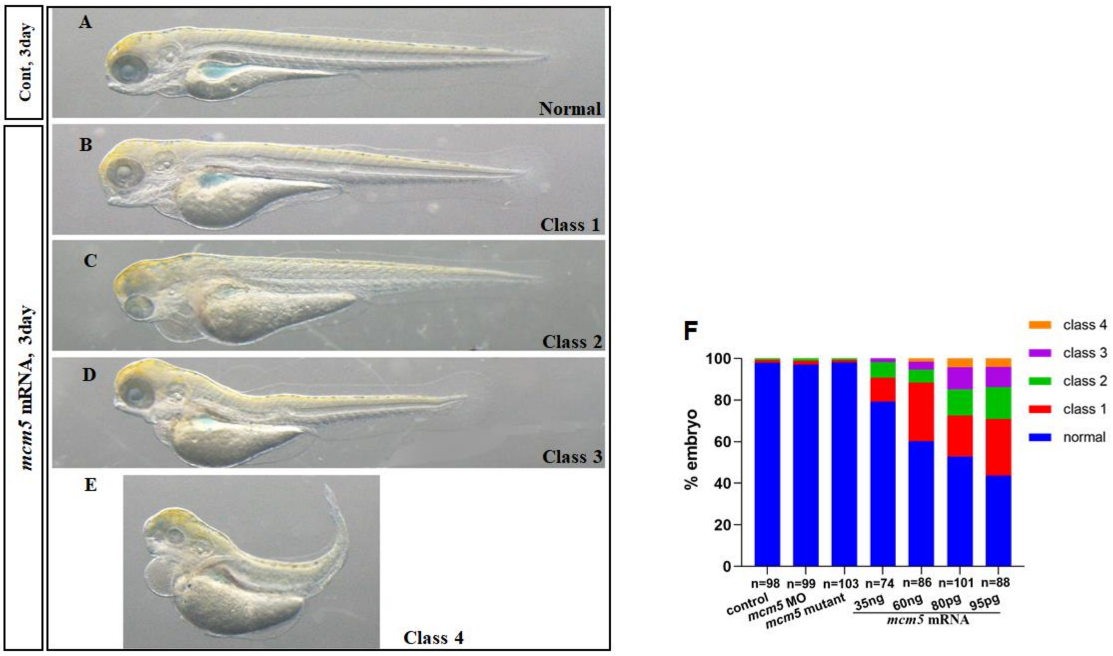

Figure S3. The overall phenotype of the embryos injected with different mRNA injection.The embryos were classified into 5 classes according to the overall phenotype (**A**–**E**). Control embryos (95.9%, *n* = 98), *mcm5* morphants(94%, *n* = 99) and *mcm5* mutants (97%, *n* = 103)displayed normal development (**F**). Different dosage of *mcm5* mRNA was used to injected at one cell stage (**F**, column 4–7), 80 pg of *mcm5* mRNA injection will lead to 28% of embryos develop abnormally (**C**, **D**, class 2–4; F, column 6, 7), 60pg or less *mcm5* mRNA injection only resulted in 15% of embryos or less developed abnormally (A-E; F, column 4 and 5).

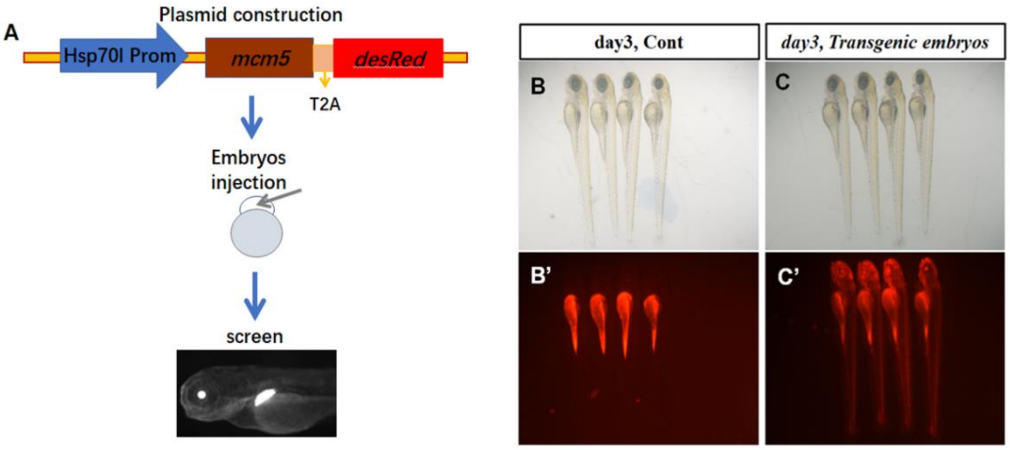

Figure S4. The construction of *Tg(Hsp70l:mcm5-T2A-desRed)* and overexpression of mcm5 by heat-shock inducing. (**A**) To generate the plasmid for generating *Tg(Hsp70l:mcm5-T2A-desRed)* transgenic line, Hsp70l promoter, the CDs of *mcm5* and T2A-desRed sequence were ligated into the main transgenic plasmid, in which the eye marker was used in the main construction. After getting the right construction, it was injected and the embryos were fed up and screened out the offspring (**A**). The transgenic embryos and the control embryos were heat-shocked on 2dpf, on 3dpf *mcm5* was successfully induced expression in transgenic embryos (**C**, **C′**，*n* = 206) while not in control embryos (**B**, **B′**，*n* = 52).

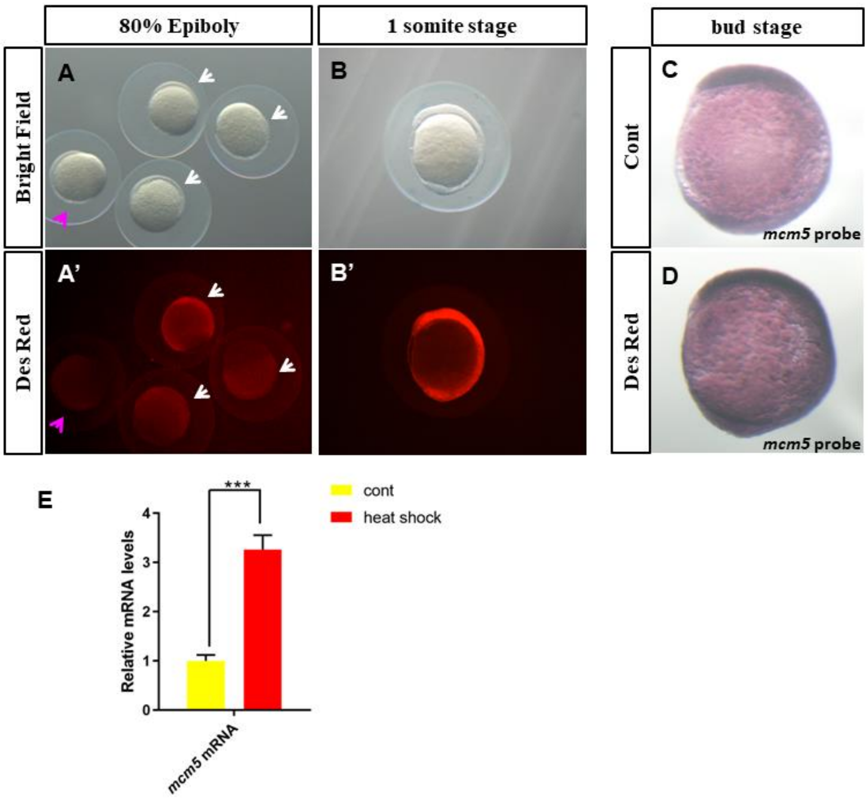

Figure S5. Heat-shock induced the expression of *mcm5* in *Tg(Hsp70l:mcm5-T2A-mCherry)* embryos. (**A**–**B′**) After being treated with heat-shock for 30 minutes at 40% epiboly, the Red Fluorescence was observed in the transgenic embryos (**A**, **A′**, white arrow showed, *n* = 86) while not in controls (**A**, **A′**, pink arrow showed, *n* = 31). At 1 somite stage, the Fluorescence

is stronger than that in early stage embryos (**B**, **B′**, *n* = 128). (**C**–**D**) *In situ* staining for the *mcm5* probe showed that, comparing with controls (**C**, *n* = 31), the transcription of *mcm5* was up-regulated in transgenic embryos (**D**, *n* = 35). (**E**) qRT-PCR showed that the relative level of *mcm5* mRNA in heat-shocked embryos was 2.26 folds to that in control embryos ($p < 0.001$).

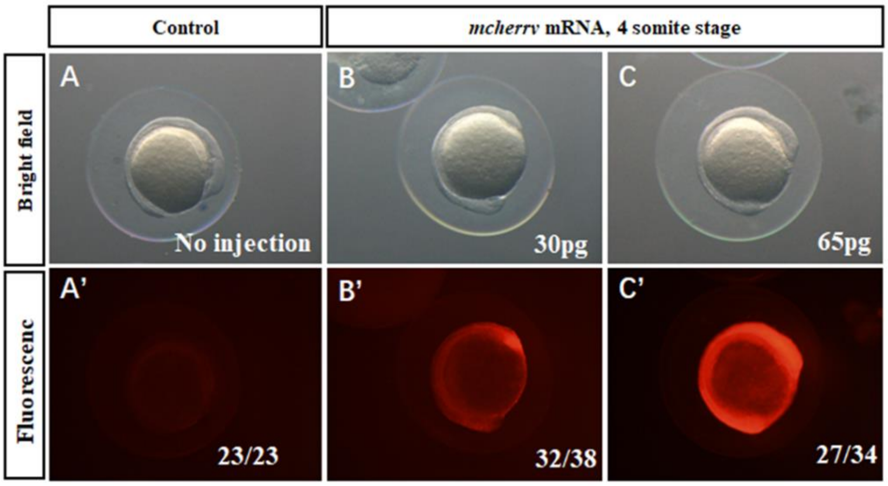

Figure S6. The expression of *mCherry* after injection of *mCherry* mRNA. (**A**–**C**) Overexpression of *mCherry* in early embryos. In control embryos, red Fluorescence was not observed (**A**, **A′**, *n* = 23). In the embryos injected with *mCherry* mRNA, the red Fluorescence was observed (**B**, **B′**, *n* = 38; **C**, **C′**, *n* = 34).

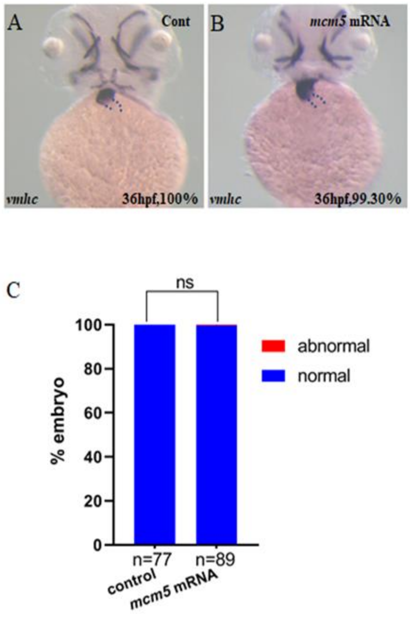

Figure S7. *mcm5* mRNA injection didn't lead to heart bifida. (**A**–**C**) At 36hpf, *vmhc* staining showed that 100% of control embryos displayed D-Loop heart (**A**, **C**; *n* = 77). forced expression of *mcm5* mRNA didn't resulted in heart developmental

defects, with normal heart while not heart bifida (**B**, **C**; *n* = 89).

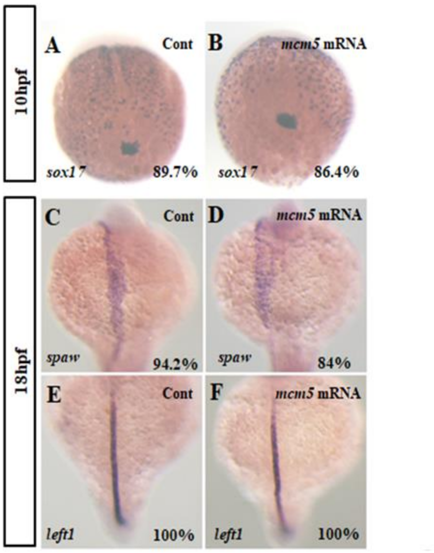

Figure S8. The expression of left-right patterning related genes. (**A**–**B**) Comparing with the control (**A**, *n* = 78), the expression of *sox17* in DFCs was not changed in majority of embryos injected with *mcm5* mRNA (B, *n* = 81). (**C**–**F**) The expression pattern of *Nodal/spaw* and nodal downstream gene *left1* were normal in control embryos (**C**, *n* = 52; **E**, *n* = 31) and majority of embryos forced expression of *mcm5* mRNA (**D**, *n* = 44; **F**, *n* = 26).

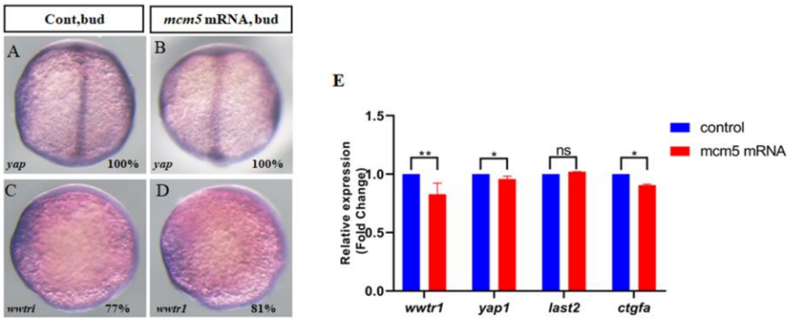

Figure S9. Yap signaling was not disturbed greatly in embryos injected with *mcm5* mRNA. (**A**–**D**) Compared with control embryos (**A**, *n* = 30), the expression of yap was mild down-regulated in embryos injected with *mcm5* mRNA (**B**, *n* = 22). *Wwtr1* expression was also mild down-regulated (**C**, *n* = 26; D, *n* = 21). (**E**) qRT-PCR showed that *wwtr1* (0.83 folds to control, $p < 0.01$), *yap1* (0.96 folds to control, $p < 0.05$) and *ctgfa* (0.91 folds to control, $p < 0.05$) were all mild down-regulated, but the expression of *last2* was not changed ($p > 0.05$).

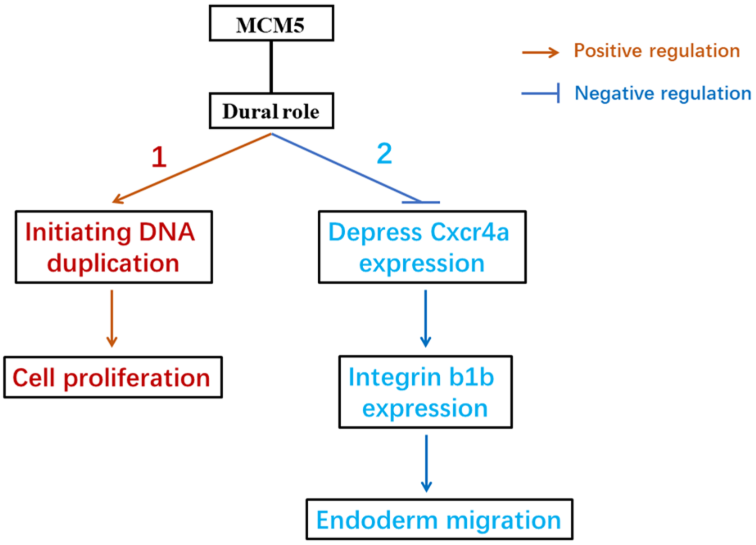

Figure S10. The model for *mcm5* regulating DNA replication and endodermal migration in early zebrafish development. During gastrulation in zebrafish, most of cells are in cell cycle with high proliferation potentiality. Mcm5 plays a dual role in this process. On the one hand, *mcm5* works as the cell cycle regulator to initiate DNA replication, ensuring DNA integrate during cell proliferating (as shown in role "1"). On the other hand, via a cell cycle independent way, *mcm5* works as a critical gene transcription regulator to directly/indirectly repress *cxcr4a* expression, sequentially regulate *itgb1b* expression and endodermal migration (as shown in role "2").

Table S1. Primers for qRT-PCR.

Gene Name	Forward Primer	Reverse Primer
cxcr4a	5′-gctggagaagtcttttagagatg-3′	5′-ctggcaacccattaccagaac-3′
itgb1b	5′-cagttctgcttggactcagcag-3′	5′-caggctggttcttgacgatgg-3′
itgb1a	5′-catcatcagctgcagtcgtgc-3′	5′-cactggtgtattcttcaggattctc-3′
cxcl12b	5′-ctcttggcatggatagcaaagtag-3′	5′-ctttgggttgatgcagacctc-3′
beta actin	5′-cccagacatcagggagtg-3′	5′-tctctgttggctttgggatt-3′
yap1	5′-acatcatgaacccagcctcag-3′	5′-tgctggttcattgcgaaacg-3′
wwtr1	5′-cgcagtctttcttccaggag-3′	5′-tgccatgtggtgatcttctc-3′
lats2	5′-tccgatggactcacaactca-3′,	5′-agcatctcaaacaggatcac-3′
ctgfa	5′-ctgcacagccagagatg-3′	5′-cacttcccaggcacttt-3′
mcm5	5′-cgatcgcactggattcacctac-3′	5′-gaggacgtgtgacctcatcag-3′
